# Transcriptomic Changes Resulting From *STK32B* Overexpression Identify Pathways Potentially Relevant to Essential Tremor

**DOI:** 10.3389/fgene.2020.00813

**Published:** 2020-07-31

**Authors:** Calwing Liao, Faezeh Sarayloo, Veikko Vuokila, Daniel Rochefort, Fulya Akçimen, Simone Diamond, Gabrielle Houle, Alexandre D. Laporte, Dan Spiegelman, Qin He, Hélène Catoire, Patrick A. Dion, Guy A. Rouleau

**Affiliations:** ^1^Department of Human Genetics, McGill University, Montreal, QC, Canada; ^2^Montreal Neurological Institute, McGill University, Montreal, QC, Canada; ^3^Department of Biomedical Sciences, Université de Montréal, Montreal, QC, Canada; ^4^Department of Neurology and Neurosurgery, McGill University, Montreal, QC, Canada

**Keywords:** *STK32B*, essential tremor, transcriptome, *FUS*, neurology

## Abstract

**Objective:** Essential tremor (ET) is a common movement disorder that has a high heritability. A number of genetic studies have associated different genes and loci with ET, but few have investigated the biology of any of these genes. *STK32B* was significantly associated with ET in a large genome-wide association study (GWAS) and was found to be overexpressed in ET cerebellar tissue. The objective of this study is to determine the effects of overexpressed *STK32B* in cerebellar DAOY cells.

**Methods:** Here, we overexpressed *STK32B* RNA in human cerebellar DAOY cells and used an RNA-Seq approach to identify differentially expressed genes (DEGs) by comparing the transcriptome profile of these cells to one of the control DAOY cells.

**Results:** Pathway and gene ontology enrichment identified axon guidance, olfactory signaling, and calcium-voltage channels as significant. Additionally, we show that overexpressing *STK32B* affects transcript levels of previously implicated ET genes such as *FUS*.

**Conclusion:** Our results investigate the effects of overexpressed *STK32B* and suggest that it may be involved in relevant ET pathways and genes.

## Introduction

Essential tremor (ET) is one of the most common movement disorders and is typically characterized by a kinetic tremor in the hand or arms (Elble, 2013). The severity tends to increase with age and may involve different regions such as the head, voice, or jaw (Haubenberger and Hallett, 2018). Previous studies investigating the histology of post-mortem tissue have pinpointed the cerebellum as a region of interest for ET (Gironell, 2014). Specifically, abnormalities with Purkinje cell axons and dendrites were found in ET brains (Axelrad et al., 2008). Furthermore, the olivocerebellar circuitry has been implicated in ET pathology and many calcium voltage channels are highly expressed in this circuitry (Schmouth et al., 2014).

The genetic etiology of ET has remained largely elusive and most studies have focused on common or rare variants and on looking for genetic overlap with other disorders (Houle et al., 2017; Liao et al., 2018). Twin studies have shown that ET has a concordance of 69–93% in monozygotic twins and 27–29% in dizygotic twins, which suggests that both genetic and environmental factors drive the onset and development of this complex trait (Tanner et al., 2001). A recent genome-wide association study (GWAS) identified a significant locus in *STK32B* and found that ET patients overexpressed *STK32B* in cerebellar tissue by comparison to healthy controls, suggesting potential implications of *STK32B* in ET (Müller et al., 2016). *STK32B* is transcribed and translated into YANK2, a serine/threonine kinase, that has not been well characterized. There have been several exome-wide studies that implicated different genetic variants as causes of ET. The first ET-implicated gene found through exome sequencing was the Fused in Sarcoma gene (*FUS*) (Merner et al., 2012). However, it is unclear whether these “ET” genes interact or have indirect effects on the expression of each other.

To understand the effects of overexpressed *STK32B* mRNA and identify pathways potentially relevant to ET, we overexpressed this gene in human cerebellar DAOY cells and compared transcriptomic changes in overexpressed cells and empty-vector controls using RNA sequencing. Several interesting pathways such as axon guidance, calcium ion transmembrane transport, and olfactory transduction were significantly enriched after overexpression of *STK32B*. We also identified previously implicated ET genes whose expression is dysregulated through the overexpression of *STK32B*, suggesting that overexpressed *STK32B* may have relevant downstream effects.

## Results

### Confirming Overexpression of *STK32B*

**FIGURE 1:**
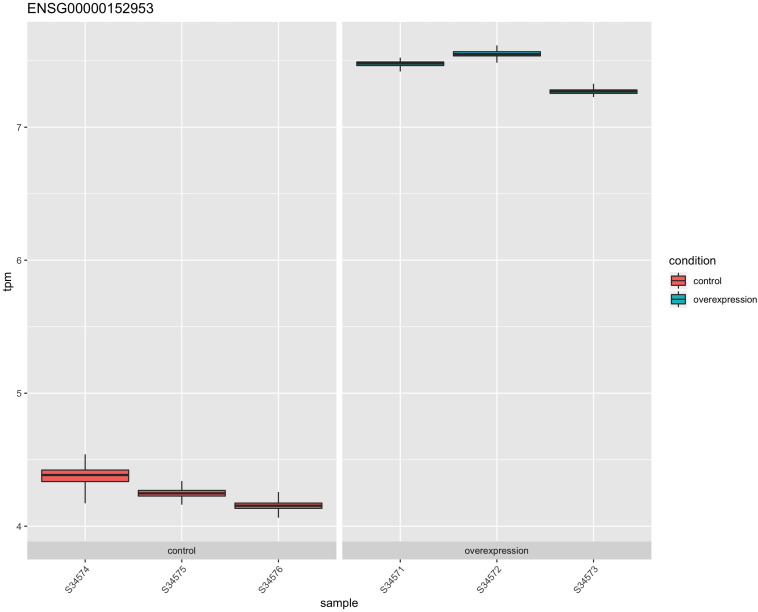
The RNA expression of *STK32B* overexpressed cells compared to empty-vector controls based on RNA sequencing data. Error bars show the technical variance determined with 200 bootstraps. TPM, transcript per million.

**FIGURE 2:**
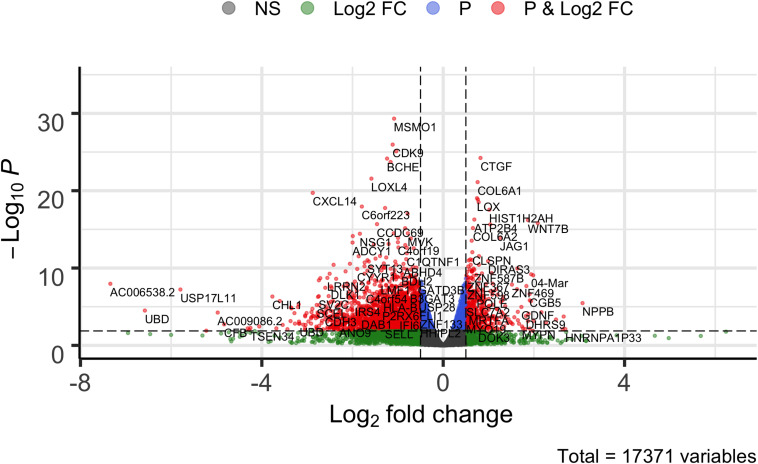
Volcano plot of comparing overexpressed *STK32B* to empty-vector controls. Differentially expressed genes (Wald test, *P*_FDR–corrected_ < 0.05 and β > | 0.05|) are shown in red. NS, not significant; FC, fold change.

The *STK32B* stable cell lines had higher RNA expression of *STK32B*, which was detected by reverse-transcriptase qPCR (Supplementary Figure S1). RNA sequencing (RNA-Seq) also confirmed that the stable cell lines have higher *STK32B* RNA levels compared to controls (Figures 1, 2). The *STK32B* gene was the top significant differentially expressed gene (*P* = 7.07E-245, β = 3.1789).

### Quality Control of RNA-Seq Analyses

**FIGURE 3:**
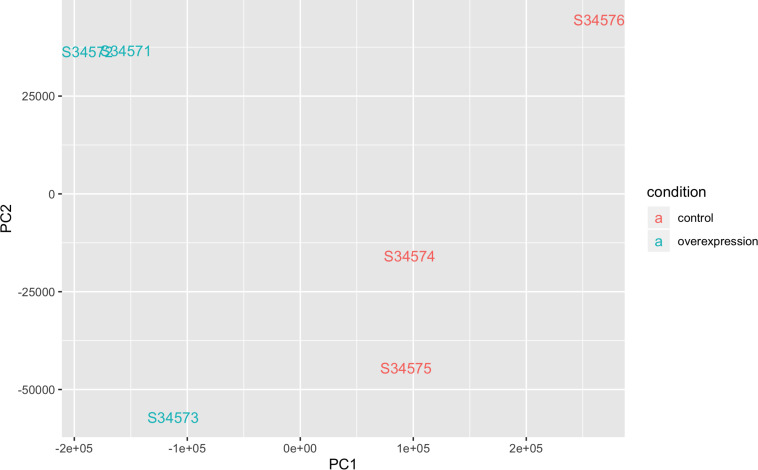
Principle component analysis plot of RNA sequencing data of *STK32B* overexpressed cells and empty-vector controls. Overexpressed cells shown in blue and controls shown in red.

The QQ-plot for differentially expressed genes (DEGs) did not show stratification or potential biases (Supplementary Figure S2). The M-A plot showed slight enrichment of downregulated genes compared to upregulated genes (Supplementary Figure S3). A principal component (PC) plot showed separation of controls and *STK32B* cell lines (Figure 3). Additionally, the dendrogram with heatmap shows that controls and *STK32B* cell lines are distinct (Supplementary Figure S4). The mean-variance plot shows the shrinkage under the sleuth model (Supplementary Figure S5).

### *STK32B* Overexpression Affects the Expression of Previously Identified ET-Associated Genes

A total of 3,794 genes were found to be differentially expressed with a *q*-value < 0.05. There were 425 genes with a β > | 0.5| and *q*-value < 0.05. Amongst the 425 genes, there were several potentially relevant ET genes were dysregulated such as *FUS* (*q*-value = 0.007, β = 0.812) and two calcium voltage channel genes that are enriched in the olivocerebellar circuitry, *CACNA1C* (*q*-value = 0.011, β = 0.503) and *CACNA1A* (*q*-value = 0.030, β = 0.702) (Table 1). Several pathways and gene ontology terms were enriched for DEGs due to the overexpression of *STK32B*, including olfactory transduction (*P* = 2.68E-39), axon guidance (*P* = 9.50E-36), and calcium ion transmembrane transport (*P* = 3.02E-31) (Table 2).

**TABLE 1:** Effects of *STK32B* perturbation on ET-implicated genes.

**Genes**	***P*-value**	***Q-value***	**Beta**
*MTHFR*	3.22E-15	2.15E-12	–1.0905696
*SHF*	6.74E-07	2.72E-05	–0.9995849
*MAPT*	2.15E-06	6.81E-05	–1.3021309
*PPARGC1A*	6.87E-06	0.00016336	–1.691415
*SNCA*	0.00071236	0.0054543	–0.4253643
*FUS*	0.00090877	0.00653715	0.81221158
*CACNA1C*	0.001691143	0.010366605	0.503658389
*PPP2R2B*	0.00168856	0.01035793	–0.7621076
*GSTP1*	0.00249219	0.01388975	–0.2723091
*GBA*	0.00326972	0.01712692	–0.4716877
*CACNA1A*	0.00306972	0.03713307	0.70202999
*LINGO1*	0.02206061	0.07248666	–1.2292913
*HNMT*	0.05546041	0.14360684	–0.3656343
*LRRK1*	0.07843181	0.18520013	0.30296973
*VDR*	0.07963403	0.18738651	0.26752548
*MAPT*	0.08202636	0.19175911	–1.786066
*HMOX1*	0.08258822	0.19278723	0.28335731
*ALAD*	0.09508151	0.21263593	–0.2562971
*LRRK2*	0.20628994	0.36752893	–0.3644185
*MAPT*	0.2395589	0.40824182	–1.0324492
*NOS1*	0.26006033	0.43219823	–1.558382
*HMOX2*	0.30661818	0.48353042	–0.2005729
*IL1B*	0.65088456	0.78183136	0.19430951
*SLC1A2*	0.66644945	0.79215448	–0.2943051

**TABLE 2:** Gene ontology and pathway analyses of differentially expressed genes.

**Gene set ID**	**Gene set name**	***P***	**Database**
KEGG_OLFACTORY_ TRANSDUCTION	Olfactory transduction	2.68E-39	KEGG
KEGG_PATHWAYS_ IN_CANCER	Pathways in cancer	2.96E-28	
KEGG_FOCAL_ ADHESION	Focal adhesion	1.19E-26	
KEGG_BLADDER_ CANCER	Bladder cancer	6.53E-25	
KEGG_ECM_ RECEPTOR_ INTERACTION	ECM-receptor interaction	8.38E-25	
GO:0001650	Fibrillar center	1.13E-29	GO Cellular
GO:0031012	Extracellular matrix	8.57E-27	
GO:0001725	Stress fiber	1.24E-26	
GO:0005925	Focal adhesion	2.82E-23	
GO:0015629	Actin cytoskeleton	4.02E-22	
REACTOME:R-HSA-381753	Olfactory signaling pathway	3.22E-46	Reactome
REACTOME:R-HSA-418555	G alpha (s) signaling events	1.19E-29	
REACTOME:R-HSA-3000170	Syndecan interactions	1.53E-27	
REACTOME:R-HSA-2022090	Assembly of collagen fibrils and other multimeric structures	4.31E-25	
REACTOME:R-HSA-1474290	Collagen formation	2.35E-24	
GO:0050911	Detection of chemical stimulus involved in sensory perception of smell	1.00E-44	GO Processes
GO:0007411	Axon guidance	9.50E-36	
GO:0048146	Positive regulation of fibroblast proliferation	1.31E-33	
GO:0070588	Calcium ion transmembrane transport	3.02E-31	
GO:0050907	Detection of chemical stimulus involved in sensory perception	3.14E-31	

### Gene Network Analyses

To identify which cluster of DEGs drove the significant pathways, gene network analysis based on co-expression was done using and identified 14 different clusters within the DEGs (Supplementary Table S1). Analysis of the top 3 largest clusters for pathway enrichments found several similar pathways in different databases including axon guidance (*P* < 1.09E-12) in cluster 1 and calcium signaling pathway in cluster 3 (2.06E-05).

## Discussion

The genetic etiology of ET is complex and likely explained by a combination of copy number variants, rare variants, gene-gene interactions and common variant drivers. One of the most significantly enriched pathways we identified was olfactory signaling and transduction. Previous reports have shown conflicting evidence about olfactory loss in ET (Applegate and Louis, 2005; Shah et al., 2008). It may be that ET patients with dysregulated olfactory signaling have overexpressed *STK32B*, which may contribute to the subset of ET patients with olfactory loss.

Interestingly, *FUS* was amongst the genes dysregulated when *STK32B* was overexpressed. In the exome familial ET study that identified *FUS*, we also found reduced mRNA levels for *FUS* (Merner et al., 2012). Similarly, the expression of *FUS* is lower when *STK32B* is overexpressed, suggesting an indirect relationship between the two genes. Additionally, two calcium voltage-gated channel genes, *CACNA1C* and *CACNA1A* were overexpressed. Enrichment analyses found the GO term calcium ion transmembrane transport to be highly significant in GO Biological Processes, suggesting that *STK32B* may play a role upstream of these genes. In the olivocerebellar circuitry, a system implicated in ET, is enriched for both of these genes (Mori et al., 1991; Schmouth et al., 2014). *CACNA1A* has been shown to be predominantly expressed in Purkinje cells, a cell type relevant to ET (Mori et al., 1991). Another enriched pathway was axon guidance. A previous study identified the *TENM4* missense variant segregating within ET families (Hor et al., 2015). *TENM4* is a regulator of axon guidance and myelination and Tenm4 knockout mice showed an ET phenotype, suggesting that axon guidance is important in ET (Hor et al., 2015). Similarly, overexpressed *STK32B* could be dysregulating important pathways relevant to axon guidance.

By sub-stratifying the overexpressed genes into different clusters that are co-expressed, several interesting pathways involving the cardiovascular system were identified. GO terms and pathways such as right ventricular cardiomyopathy in KEGG and angiogenesis in GO Biological Processes were found among the significant pathways in Table 3. In certain ET patients, beta-blockers can reduce tremor magnitude and frequency. Beta-blockers lower blood pressure and are used to treat irregular heart rhythm and other cardiovascular phenotypes. A common treatment for ET is propranolol, a beta-blocker. This was a drug developed for cardiovascular health that affects adrenergic activity, but still reduces tremor in ET individuals. This could suggest that overexpressed *STK32B* affects cardiovascular health, which may in turn affect or lead to the ET phenotype or that *STK32B* may be pleiotropic and affect the nervous and cardiovascular system differently.

**TABLE 3:** Gene ontology and pathway analyses of differentially expressed gene for the three largest clusters based on co-expression.

**Gene set name**	***P*-value**	**Database**	**Cluster**
Regulation of actin cytoskeleton	1.03E-13	KEGG	1
Pathways in cancer	1.23E-13		
Axon guidance	1.09E-12		
Focal adhesion	4.96E-12		
Arrhythmogenic right ventricular cardiomyopathy (ARVC)	4.63E-09		
Fibrillar center	1.13E-29	GO Cellular	
Extracellular matrix	8.57E-27		
Stress fiber	1.24E-26		
Focal adhesion	2.82E-23		
Actin cytoskeleton	4.02E-22		
Actin binding	3.66E-15	GO Function	
Laminin binding	2.37E-13		
Insulin-like growth factor binding	9.72E-13		
Collagen binding	3.73E-12		
Protein heterodimerization activity	5.54E-12		
Cell migration involved in sprouting angiogenesis	7.07E-18	GO Processes	
Cell migration	2.94E-16		
inactivation of MAPK activity	3.31E-15		
Cytoskeleton organization	6.34E-15		
Negative regulation of cell migration	9.12E-15		
Cell-extracellular matrix interactions	7.16E-15	Reactome	
Signal transduction	1.98E-13		
Non-integrin membrane-ECM interactions	2.58E-12		
Signaling by Rho GTPases	1.84E-11		
EPHA-mediated growth cone collapse	2.30E-11		
RNA polymerase	4.57E-13	KEGG	2
Pyrimidine metabolism	1.37E-12		
Alzheimer’s disease	3.55E-11		
Purine metabolism	4.11E-11		
GnRH signaling pathway	1.84E-09		
Nucleolus	5.96E-19	GO Cellular	
Preribosome, large subunit precursor	2.21E-18		
Small-subunit processome	5.13E-18		
DNA-directed RNA polymerase III complex	5.10E-15		
DNA-directed RNA polymerase I complex	1.85E-14		
Pseudouridine synthase activity	7.75E-16	GO Function	
RNA binding	1.25E-15		
snoRNA binding	4.90E-14		
rRNA binding	3.55E-13		
ATP-dependent RNA helicase activity	5.23E-13		
RNA secondary structure unwinding	1.57E-16	GO Processes	
tRNA modification	7.73E-16		
Positive regulation of gene expression, epigenetic	3.09E-15		
Ribosomal large subunit biogenesis	3.17E-15		
Maturation of SSU-rRNA from tricistronic rRNA transcript (SSU-rRNA, 5.8S rRNA, LSU-rRNA)	3.32E-15		
rRNA modification in the nucleus and cytosol	3.38E-18	Reactome	
tRNA processing	1.65E-17		
tRNA modification in the nucleus and cytosol	1.66E-16		
rRNA processing	9.91E-15		
tRNA processing in the nucleus	1.69E-14		
RIG-I-like receptor signaling pathway	1.24E-10	KEGG	3
Cytosolic DNA-sensing pathway	1.92E-07		
O-glycan biosynthesis	1.163E-06		
Focal adhesion	1.36893E-06		
Calcium signaling pathway	2.06986E-05		
Caveola	2.00E-08	GO Cellular	
Proteinaceous extracellular matrix	7.41E-08		
Postsynaptic membrane	2.45E-06		
Microfibril	4.99E-06		
Extracellular matrix	1.12E-05		
Type I interferon receptor binding	3.64E-07	GO Function	
Neuropeptide binding	5.89E-07		
Scavenger receptor activity	6.23E-07		
Calcium-dependent phospholipid binding	9.44E-07		
RNA binding	2.46E-06		
Response to mechanical stimulus	8.42E-09	GO Processes	
Lung development	1.55E-08		
Negative regulation of neuron apoptotic process	5.29E-08		
Cellular response to hypoxia	7.29E-08		
Regulation of type I interferon-mediated signaling pathway	1.11E-07		
O-glycosylation of TSR domain-containing proteins	3.73E-10	Reactome	
Defective B3GALTL causes Peters plus syndrome (PpS)	5.15E-10		
DDX58/IFIH1-mediated induction of interferon-alpha/beta	1.29E-09		
O-linked glycosylation	4.91E-08		
Diseases associated with O-glycosylation of proteins	1.04E-07		

Due to the heterogeneity of ET, it is likely that not all ET-affected individuals have overexpression of *STK32B*. One limitation of this study is that DAOY cells are a cancerous cell line and may not be the ideal model for understanding the transcriptome of Purkinje cells. Future studies on the effects of *STK32B* overexpression could use induced pluripotent stem cells differentiated into cells with Purkinje cells properties; such cells would likely have less biological noise. However, our findings support the idea that *STK32B* is a gene of interest for a subset of ET cases, and further investigations into how *STK32B* may interact with other genes are warranted.

## Materials and Methods

### Cell Culture and Plasmid Construction

The DAOY cell line was cultured in Eagle’s Minimum Essential Medium (EMEM) with 10% fetal bovine serum (FBS) at 37°C and 5% CO_2_ with the Glico Pen-Strep-Glutamine cocktail at 1×. Cells were passaged every 2 days at 80–90% confluence and at the same time. The cDNA of *STK32B* (NM_001306082.1) was inserted into a pcDNA3.1(+) vector containing a neomycin resistance gene, used as a selectable marker. This transcript was picked because it had the highest expression in the cerebellum based on GTEx 53 v7. The plasmid was transformed and expanded in XL10-Gold *Escherichia coli* strain (Stratagene). The cDNA of the plasmid was sent for Sanger sequencing to validate the gene sequence.

### Transfection and Deriving Stable Cell Lines

The cells were transfected for 48 h with 8 ug of the vector of using the jetPRIME transfection reagent (Polyplus). A green fluorescent protein (GFP) control vector was used to assess and estimate transfection efficiency and optimize transfection parameters. Stable cell lines were established by subjecting transfected cells to G418 antibiotic at 1 μg/mL for 10 days and maintained at a concentration of 400 μg/mL for an additional 2 weeks. A kill curve was done to determine the ideal antibiotic selection. In parallel, empty pcDNA3.1(+) vector control lines were also grown and underwent antibiotic selection.

### RNA Extraction and Sequencing

Total RNA was extracted using the RNeasy Mini Kit (Qiagen). The total RNA concentration was measured using the Synergy H4 microplate reader. RNA for the top three overexpressed *STK32B* cell lines and three empty-vector controls was sent to Macrogen Inc., for sequencing. RT-qPCR was used to confirm overexpression in the cell lines compared to controls. Preparation of the cDNA library was done with the TruSeq Stranded Total RNA Kit (Ilumina) with Ribo-Zero depletion. Sequencing was done on the NovaSeq 6000 at 150 bp paired-end reads with a total of 200M reads. Samples were randomized for cell lysis, RNA extraction, Ribo-Zero depletion, library preparation and sequencing to account for potential batch effects.

### Data Processing and Quality Control

The FASTQ files were pseudo-aligned with Salmon using the Ensembl v94 annotation of the human genome (Patro et al., 2017). The data is available on the public repository of NCBI, Gene Expression Omnibus (GEO), with the GEO accession as GSE150393. It can be accessed with the following URL: https://www.ncbi.nlm.nih.gov/geo/query/acc.cgi?acc=GSE150393. The parameters of Salmon included the following: 200 bootstraps with mapping validation, GC bias correction, sequencing bias correction, 4 range factorization bins, a minimum score fraction of 0.95 and VB optimization with a VB prior of 1e-5. The estimated counts were analyzed using sleuth to identify DEGs (Pimentel et al., 2017). A Wald test was used to get β values [log_2_(x + 0.5)]. Additionally, transcript-level aggregation was done against the Ensembl v94 annotation to determine gene-level differential expression. *P*-values were corrected using the Benjamini–Hochberg procedure to account for false discovery rate (FDR). The FDR threshold was set at 0.05. A PC plot and heatmap was done to identify clusters.

### Pathway and Gene Set Enrichment Analysis

Pathway and gene set enrichment for relevant databases was done using Gene Network 2.0 (Deelen et al., 2019). To investigate genes with larger fold changes, only DEGs with a β > |0.5| were analyzed for pathway enrichments. Network clustering was done by grouping genes based on co-expression using Gene Network 2.0. Pathway analyses were also conducted with Gene Network 2.0 for the clusters using a Wilcoxon test. We included the top five significant pathways across different databases.

## Data Availability Statement

The data is available on the public repository of NCBI, Gene Expression Omnibus (GEO), with the GEO accession as GSE150393. It can be accessed with the following URL: https://www.ncbi.nlm.nih.gov/geo/query/acc.cgi?acc=GSE150393.

## Ethics Statement

The review board at the McGill University Health Center Research Ethics Board (MUHC REB) approved the study protocols (reference number IRB00010120).

## Author Contributions

CL conducted the experiments and analyses and drafted the manuscript. FS, VV, DR, FA, SD, GH, QH, and HC helped with experiments. AL and DS helped with bioinformatic analyses. PD and GR oversaw the study and drafting of the manuscript. All authors contributed to the article and approved the submitted version.

## Conflict of Interest

The authors declare that the research was conducted in the absence of any commercial or financial relationships that could be construed as a potential conflict of interest.
