# Supplementary material for: Transcriptomic Changes Resulting From STK32B Overexpression Identify Pathways Potentially Relevant to Essential Tremor
Source: Front Genet. 2020 Jul 31;11:813. doi: 10.3389/fgene.2020.00813 (PMC7413243; doi:10.3389/fgene.2020.00813)
Supplement: Supplementary file 2 [file Presentation_1.pdf]

Supplementary Figures

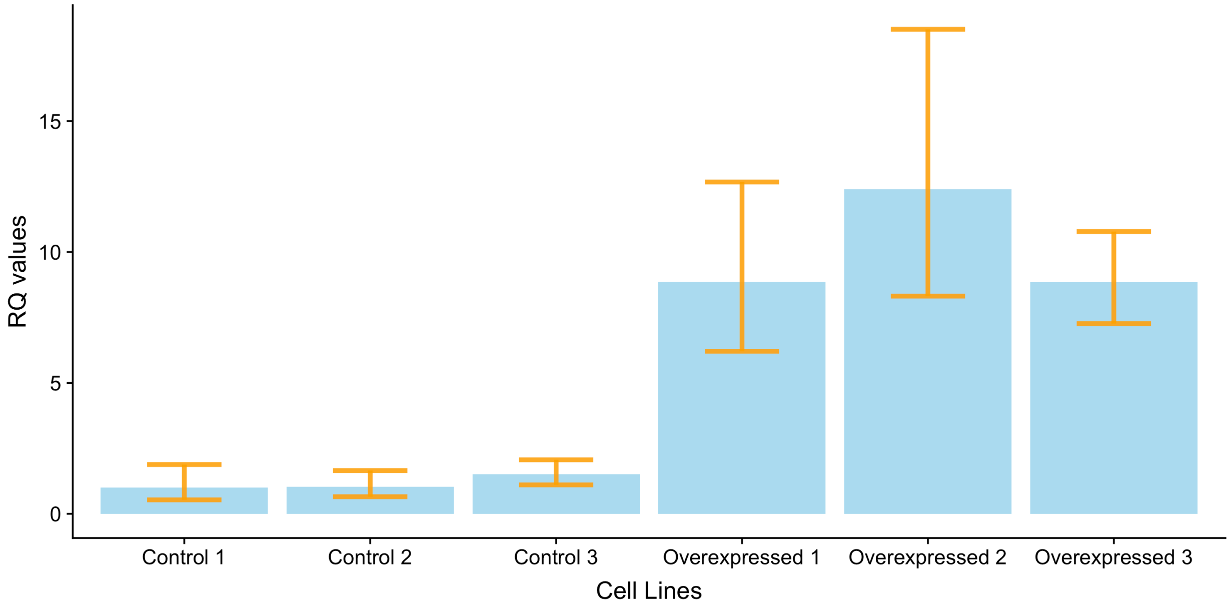

Supplementary Figure 1. Relative quantification values of RNA sent for sequencing. Samples were done in quadruplicate. Error bars represent min and max amongst the quadruplicates.

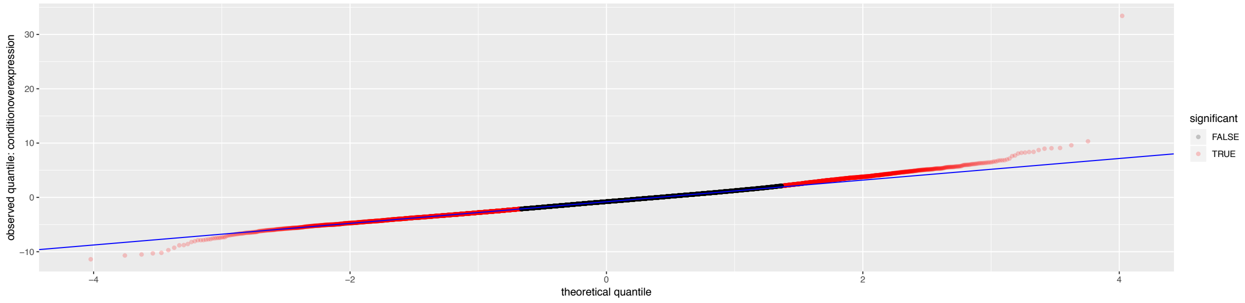

Supplementary Figure 2. QQ-plot of differentially expressed genes for RNA sequencing data.

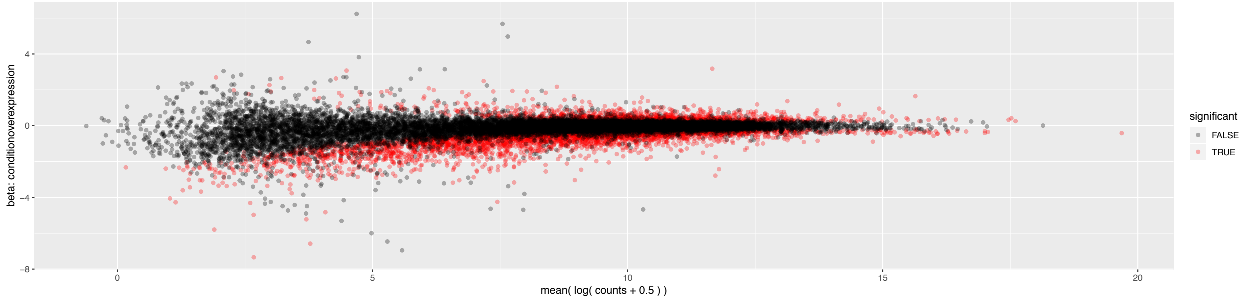

Supplementary Figure 3. MA-plot of RNA sequencing data.

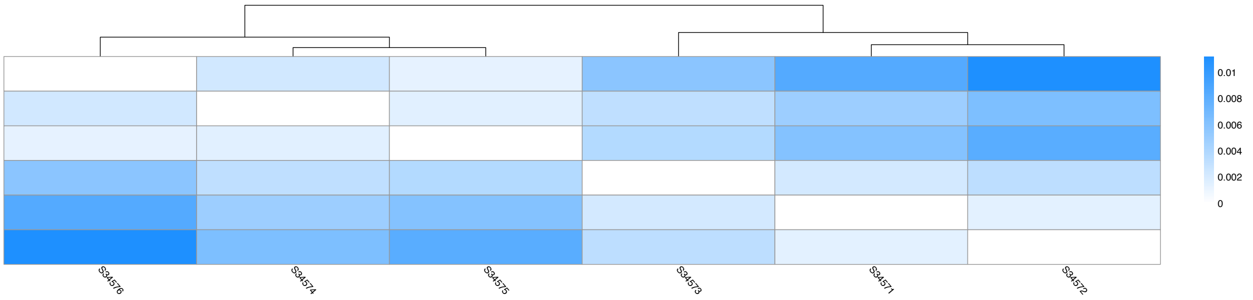

Supplementary Figure 4. Heatmap of RNA sequencing data for both controls and overexpressed. S34571, S34572 and S34573 were overexpressed cells. S34574, S34575 and S34576 were controls.

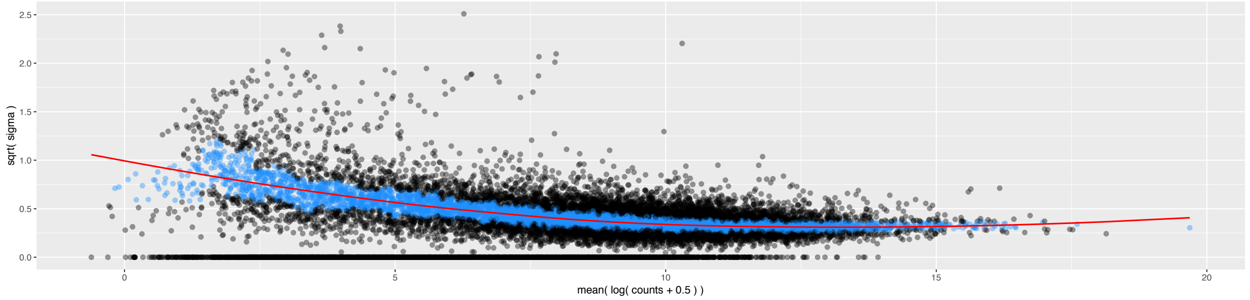

Supplementary Figure 5. Mean-variance plot of the differentially expressed data. Data was processed through sleuth for differential expression.
